# Evolution of SARS-CoV-2 IgG Seroprevalence in Children and Factors Associated with Seroconversion: Results from a Multiple Time-Points Study in Friuli-Venezia Giulia Region, Italy

**DOI:** 10.3390/children9020246

**Published:** 2022-02-12

**Authors:** Marzia Lazzerini, Simone Benvenuto, Ilaria Mariani, Giorgio Fedele, Pasqualina Leone, Paola Stefanelli, Giada Vittori, Silvana Schreiber, Alberto Tommasini, Giovanni Rezza, Egidio Barbi, Manola Comar

**Affiliations:** 1 Institute for Maternal and Child Health, IRCCS “Burlo Garofolo”, 34137 Trieste, Italy; marzia.lazzerini@burlo.trieste.it (M.L.); ilaria.mariani@burlo.trieste.it (I.M.); giada.vittori@burlo.trieste.it (G.V.); silvana.schreiber@burlo.trieste.it (S.S.); alberto.tommasini@burlo.trieste.it (A.T.); egidio.barbi@burlo.trieste.it (E.B.); manola.comar@burlo.trieste.it (M.C.); 2 Department of Medical, Surgical and Health Sciences, University of Trieste, 34127 Trieste, Italy; 3 Italian National Institute of Health, 00161 Rome, Italy; giorgio.fedele@iss.it (G.F.); pasqualina.leone@iss.it (P.L.); paola.stefanelli@iss.it (P.S.); giovanni.rezza@iss.it (G.R.)

**Keywords:** COVID-19, children, seroprevalence, IgG antibodies, Italy

## Abstract

Data on the effective burden of the SARS-CoV-2 pandemic in the pediatric population are limited. We aimed at assessing the prevalence of SARS-CoV-2 IgG antibodies in children at three subsequent time-points. The study was conducted between January 2021 and July 2021 among children referring to the Research Institute for Maternal and Child Health “Burlo Garofolo” in Trieste, a referral regional hospital in Friuli Venezia Giulia, Italy. A multivariate analysis was conducted to assess factors independently associated with seroconversion. A total of 594 children were included. Anti-SARS-CoV-2 trimeric Spike protein IgG antibodies were found in 32 (15.4%) children tested in April-May and in 20 (11.8%) in June–July 2021, compared with 24 (11.1%) of those tested in January–February 2021 (*p* = 0.37, Armitage exact test for trend over time *p* = 0.76). A subgroup analysis and a multivariate logistic regression analysis were performed considering sociodemographic, clinical, and historical variables. Three categories of children showed statistically significant increased odds of positive anti-SARS-CoV-2 IgG antibodies: children previously positive to a nasopharyngeal swab (AdjOR 15.41, 95%CI 3.44–69.04, *p* < 0.001), cohabitant with a person with an history of a previous positive nasopharyngeal swab (AdjOR 9.95, 95%CI 5.35–18.52, *p* < 0.001), and children with a foreign citizenship (AdjOR 2.4, 95%CI 1.05–5.70, *p* = 0.002). The study suggests that seroprevalence studies may be of limited help in estimating the prevalence of the COVID-19 pandemic in children. Further studies are needed to identify other markers of previous SARS-CoV-2 infection in children, such as CD4+ T cells or memory B-cells.

## 1. Background

COVID-19 has been a major public health concern worldwide since 2020, and major efforts, with a high cost for the health system and the entire society, have been made to mitigate its spread. Updated data on COVID-19 incidence and prevalence, as well as studies on the immunological response to SARS-CoV-2 in different populations, are critical to plan effective prevention and control efforts and reduce cost for the society [1,2]. Longitudinal surveys to continually monitor SARS-CoV2 seroprevalence rates in different age subgroups have been considered important to support prevention and control efforts [1,2].

Particularly in children, the lack of an accurate diagnostic tests acceptable to the wide public, as well as the high rate of asymptomatic or oligosymptomatic cases, limit the availability of reliable information on COVID-19 incidence [2,3]. On one side, mechanisms of the immune reaction to SARS-CoV-2 in the pediatric age have not been fully elucidated, and it is unclear to what extent SARS-CoV-2 IgG antibodies are a good marker of previous infection. On the other side, a better understanding of children’s susceptibility to SARS-CoV-2 infection may allow to better plan public health interventions, such as school closure. The availability of more studies on seroprevalence rate in children has been considered important to contribute to fill some of these gaps in knowledge and provide data relevant both to policy makers and researchers.

Despite the high number of infected children so far, longitudinal seroprevalence studies in the pediatric population are very limited [2,4]. This lack of studies in the pediatric population is due to multiple factors, including well-known constrains and ethical considerations when performing research in healthy children, imposing the need to reduce invasive procedures such as blood sampling to balance with low risk due to COVID-19 disease in this age group [3].

Specifically, very few studies reported on seroprevalence rates over time among children in Italy. A first single time-point country-wide evaluation conducted at the end of the first peak in summer 2020 by the Italian National Institute of Statistics (ISTAT) highlighted a very low seroprevalence rate in the age group 0–17 years, ranging from 0.1% to 1.1% in North and Central Italy [5], but was not repeated afterwards. Other few studies were conducted, at either regional or local level [6,7,8,9], consistently showing, in line with the existing literature from other countries [6,7,8,9,10,11,12,13,14,15], lower seroprevalence rates in children compared to adults. However, all seroprevalence studies conducted in children in Italy [5,6,7,8,9] only included a single time-point assessment.

In January 2021, in the context of a study called COVID-IMMUNO, we conducted in Friuli Venezia Giulia Region (FVG), in the Northeast of Italy, an assessment of SARS-CoV-2 IgG antibodies among children, revealing a seroprevalence rate of 9.5% [15]. This paper reports the results of the second and third evaluation, conducted within the same project (COVID-IMMUNO), at subsequent time-points. In each evaluation, the same methods as the previous assessment [15] were used, with the primary aim to explore changes in the SARS-CoV2 IgG seroprevalence rate among children during the second and third wave of the pandemic in FVG. The secondary aim was to assess the socio-demographic factors independently associated with an IgG seroconversion.

## 2. Materials and Methods

### 2.1. Study Design

This was a cross-sectional study, performed at multiple time points, and is reported according to the Strengthening the Reporting of Observational Studies (STROBE) in Epidemiology guidelines for cross-sectional studies [16]. The study was conducted between January 2021 and July 2021.

### 2.2. Population and Settings

Children were enrolled between January 2021 and July 2021 among those who accessed the Research Institute for Maternal and Child Health “Burlo Garofolo” in Trieste, a referral regional hospital, in FVG, and who needed a blood test for any reason. Age > 18 years, risk factors for an ongoing SARS-CoV-2 infection (i.e., typical symptoms, recent positive nasopharyngeal swab, contact with a confirmed or a suspected case in the 14 days before the blood sample, travelling abroad in the 14 days before the blood sample), partial or full anti-SARS-CoV-2 vaccination (which started in Italy for children over 11 years in June 2021), and/or immunodeficiency, either primitive or secondary, were considered exclusion criteria, together with refusal to participate to the study. Drug-induced immune suppression was pre-defined for patients receiving immunosuppressants (such as methotrexate, mycophenolate, cyclosporin, azathioprine and others), biologics (such as rituximab, infliximab, adalimumab and others), immune globulins, and/or glucocorticoids (defined as methylprednisolone 1 mg/Kg or equivalent for at least 14 days).

Cases from three time periods—corresponding to the second wave of COVID-19 epidemic in the region (first time-period, January–February 2021), the end of the third wave (second time-period, April–May 2021), and the summertime, where a low incidence of cases was reported (third time-period, June–July 2021)—were collected.

### 2.3. Data Collection Procedures

Data on relevant clinical and epidemiological characteristics of children were collected with a predefined, standardized, field-tested form, which was self-compiled by parents at time of enrollment. Data collection forms were checked in real time for internal consistency or missing data by trained personnel.

Blood samples were collected in specific test tubes, which were anonymized and sent to the Italian National Institute of Health for analysis. Anti-SARS-CoV-2 trimeric Spike protein IgG antibodies were detected through chemiluminescent immunoassay (CLIA) (Diasorin, Italy; 98.7% positive percent agreement (PPA), 95% CI 94.5–99.6%).

### 2.4. Data Analysis

A sample size of 167 children per time was needed to be able to assess a seroprevalence rate of 10%, 15%, and 20%, with 80% power using a continuity corrected one-sided 5%-level Cochran–Armitage test. 

First, a descriptive analysis was performed, grouping children in three time periods. Categorical variables were reported as absolute numbers and percentages and compared between time periods using the χ^2^ or Fisher exact test as appropriate. Continuous variables were expressed as means and standard deviations or as median and inter-quartile ranges (IQR), if not normally distributed, and compared with a *t*-test or a Wilcoxon–Mann–Whitney or a Kruskal–Wallis test, as appropriate. Bonferroni correction was used in case of multiple comparisons among groups. To test time trend of seroconversion, a Cochran–Armitage trend test was performed. Data on IgG seroprevalence rate were also calculated on a weekly basis and were plotted against the reported epidemic curve in FVG Region.

Subgroup analysis by results of a previous nasopharyngeal swab was conducted to evaluate presence of anti-SARS-CoV-2 IgG antibodies among children with or without a previous diagnosis of COVID-19.

An additional subgroup analysis was performed to assess differences in sociodemographic (sex, age, citizenship, cohabitant healthworker), clinical (comorbidities such as syndromes/genetic disorders, autoimmune diseases and in particular type 1 diabetes mellitus, obesity/overweight, prematurity, asthma/allergic disease, cerebral palsy/other neurological diseases), and historical (previous positive nasopharyngeal swab, cohabitant with a history of previous positive nasopharyngeal swab) characteristics between children presenting anti-SARS-CoV-2 Spike protein IgG antibodies and children not presenting them. Secondly, a multivariate logistic regression analysis was performed using the same sociodemographic, clinical, and historical variables as independent variables. 

The significance level was set at 0.05 (two-tailed test). Data were analyzed with STATA 14 and SAS/STAT 14.3.

### 2.5. Ethical Considerations

The COVID-IMMUNO study was approved by the Institutional Review Board of the Institute for Maternal and Child Health IRCCS Burlo Garofolo, Trieste, Italy (IRB-BURLO 06/2020 27 July 2020). Children and their parents were informed about the objective and methods of the study, and parents provided their written consent. Data were collected in an anonymous way and analyzed and reported only in aggregate form.

## 3. Results

### 3.1. Patient Enrollment

**Figure 1:**
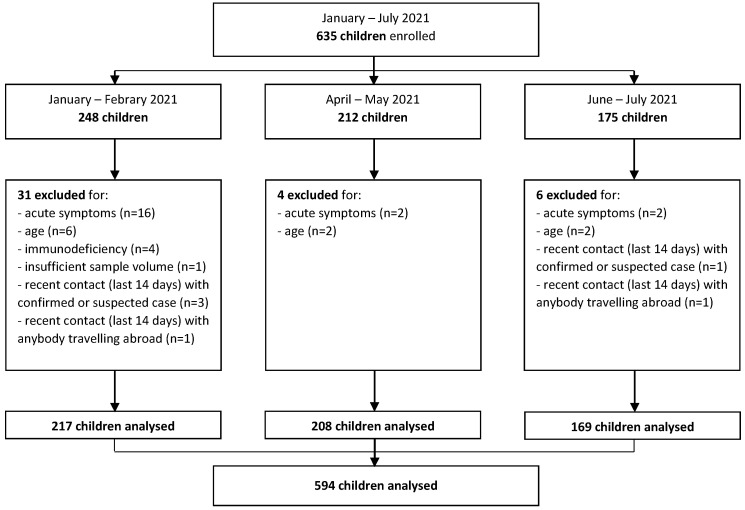
Study flow diagram.

Between January 2021 and July 2021, a total of 635 children were enrolled. Among these, after exclusion of children without inclusion criteria, 217 children were included in the analysis for the months of January–February, 208 for April–May, and 169 for June–July, for a total sample across the three time periods of 594 children (Figure 1).

### 3.2. Characteristics of Children

There were no relevant differences in baseline characteristics of children between the three groups (Table 1), except for a slightly higher prevalence of co-morbidities in the first time-period (49.8% in January–February vs. 38.0% in April–May vs. 39.1% in June–July, *p* = 0.03), with malformations/syndromes being the most prevalent comorbidity (around 11% in each group).

The median age was 11 years in each group, with all age intervals being represented, and with children ranging from 12 to 17 years old being the most prevalent age group, accounting for around 45% of the total sample. Both groups included around 8% immigrant children, as expected for the population composition in FVG [17]. Each group had a not negligible rate of cohabitant with a history of a previous positive nasopharyngeal swab (12.9% vs. 17.3% vs. 14.2%, *p* = 0.43), with a non-significant trend in time (Armitage exact test *p* = 0.67). 

In both groups, more than two-thirds of the children (68.2% vs. 71.2% vs. 74.0%, *p* = 0.46) had previously been tested with a nasopharyngeal swab, with only a small percentage (4.1% vs. 4.7% vs. 2.4%) resulting positive in each time period, with no statistically significant differences among groups (*p* = 0.59). 

### 3.3. Findings on Anti-SARS-CoV-2 IgG Antibodies

**Figure 2:**
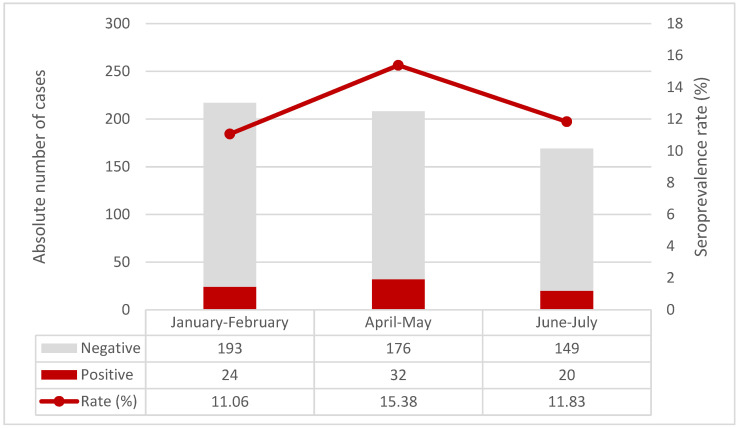
Seroprevalence rate over time in the three time periods.

**Figure 3:**
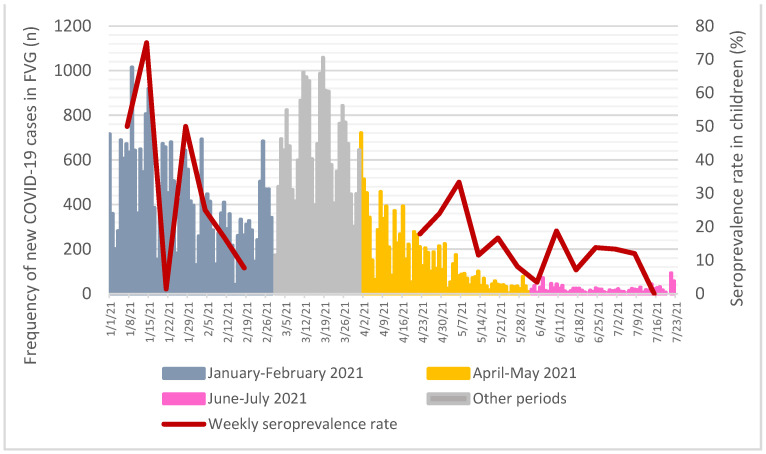
Seroprevalence rate on a weekly basis when compared to all-age new cases of COVID-19 assessed through nasopharyngeal swab in FVG Region. Data source for new COVID-19 cases: Presidency of the Council of Ministers, Civil Protection Department, data freely available at https://mappe.protezionecivile.gov.it/it/mappe-emergenze/mappe-coronavirus/situazione-desktop (accessed on 4 December 2021) [18].

Anti-SARS-CoV-2 trimeric Spike protein IgG antibodies (Table 1) were found in 32 (15.4%) children tested in April-May and in 20 (11.8%) in June–July 2021, compared to 24 (11.1%) to those tested in January–February 2021 (*p* = 0.37, Armitage exact test for trend over time *p* = 0.76, Figure 2). When compared to the COVID-19 epidemic curve in FVG Region, antibodies rates, calculated on a weekly basis, had a spike after the epidemic peaks, but then decreased rapidly (Figure 3).

### 3.4. Subgroup Analysis

In the subgroup analysis, while in the first and third time period, SARS-CoV-2 IgG antibodies were detected in all children with a history of previous SARS-CoV-2 positive nasopharyngeal swab, in the second time period three out of seven children with a previous COVID-19 diagnosis were found to have SARS-CoV-2 IgG antibodies negative. All the three cases were male adolescents, the interval between the positive nasopharyngeal swab and the antibody testing was one month for two cases and seven months in the other case, and the disease was oligosymptomatic. 

Conversely, in children with a previous negative SARS-CoV-2 nasopharyngeal swab, SARS-CoV-2 IgG antibodies were detected in 12% of children (11.3% vs. 12.1% vs. 12.3%, respectively, *p* = 0.96) (Table 2).

Characteristics of patients testing positive for anti-SARS-CoV-2 Spike protein IgG antibodies compared to those testing negative are reported in Table 3. Children presenting anti-SARS-CoV-2 IgG antibodies more often had a foreign citizenship (17.1% vs. 6.9% in children not presenting anti-SARS-CoV-2 IgG antibodies, *p* = 0.005) and a cohabitant with a history of a previous positive nasopharyngeal swab (54.0 vs. 9.1%, *p* < 0.001).

Among children ever tested with a nasopharyngeal swab (80.3% vs. 69.5%, *p* = 0.05), 21.3% resulted positive in the group with positive anti-SARS-CoV-2 IgG antibodies, whereas only three (0.8%, *p* < 0.001) were positive in the comparison group, with no statistically significant differences in the timing of performing the nasopharyngeal swab.

### 3.5. Multivariate Analysis

When adjusting for confounders in the logistic regression analysis (Table 4), differences were confirmed, with three categories of children having statistically significant increased odds of positive anti-SARS-CoV-2 IgG antibodies: children who ever resulted positive to a nasopharyngeal swab (AdjOR 15.41, 95%CI 3.44–69.04, *p* < 0.001); children with a cohabitant with a history of a previous positive nasopharyngeal swab (AdjOR 9.95, 95%CI 5.35–18.52, *p* < 0.001); and children with a foreign citizenship (AdjOR 2.4, 95%CI 1.05–5.70, *p* = 0.002).

## 4. Discussion

To date, this is the first study that performed a multiple time-point assessment of the SARS-CoV-2 seroprevalence rate in Italian pediatric population. The study fund no clinically relevant differences in the prevalence of anti-SARS-CoV-2 trimeric Spike protein IgG antibodies across three time periods: the second wave of COVID-19 epidemic in the region versus the end of the third wave (when the Delta variant was highly prevalent) and the following summertime, when a low incidence of cases was reported. Moreover, when compared to the COVID-19 epidemic curve in FVG Region, IgG antibodies rates had a spike after the epidemic peaks, but then decreased rapidly.

To our knowledge, no recent studies have evaluated anti-SARS-CoV-2 antibodies prevalence in Italy in the same period, so we lack data for a comparison. Of note, no significant differences in anti-SARS-CoV-2 IgG positivity were found in a large pediatric cohort evaluated in China over a 6-month period between March and August 2020 [19]. Data from sero-surveillance programmes implemented in England similarly showed a plateau in seropositivity in children between January and August 2021, followed by a large increase starting from September 2021, probably due to the deployment of the vaccination campaign [20]. Conversely, similar studies performed in adult cohorts consistently showed a substantial increase in seroprevalence rate of anti-SARS-CoV-2 antibodies [21,22].

The Italian COVID-19 monitoring system does not provide detailed data by region on the number of COVID cases in children [23]. Only data on all-age COVID-19 cases are available, showing that, in July 2021, about 105,000 cases occurred in our region [24], accounting for about 9% of the total population in FVG. This prevalence seems, in principle, in line with the seroprevalence rate observed in children in this study. However, the existing monitoring system may largely under-estimate the real number of COVID-19 cases due to a high frequency of asymptomatic cases and possible gaps in case finding and contact tracing [25]. On the other hand, it has been hypothesized that not all children infected with SARS-CoV-2 develop detectable antibody titers, and antibodies may quickly be volatile. In a cohort including 138 previously infected children, 25% showed a seronegative status for anti-SARS-CoV-2 IgM and/or IgG antibodies one month after the infection [26]. Moreover, previous studies on the topic have shown how antibodies titers are lower in children compared to adults [25,27], and especially in younger children when compared to adolescents [28]. Nevertheless, the observation of a higher antibody binding avidity in children compared to young adults seems to prove the effectiveness of their immune response [29]. Interestingly, a weak and delayed antibody response to SARS-CoV-2 has been associated with severe COVID-19 in children [26].

The findings of our study may have different explanations. A first explanation is that children may develop low titers of SARS-CoV-2 trimeric Spike protein IgG antibodies, which may be not always detectable by the current testing systems. A second explanation is that IgG antibodies may quickly be volatile. This seems to be in line with additional findings of our study, where three out of seven children with a PCR swab confirmed COVID-19 diagnosis tested negative to SARS-CoV-2 IgG antibodies after 1 to 7 months from the swab test. Therefore, this study suggests that seroprevalence studies may not be very helpful in estimating the prevalence of the COVID-19 epidemic in children, nor to assess previous contact with SARS-CoV-2 and their immunological response. Further studies are needed to identify other markers of previous SARS-CoV-2 infection in children.

The other interesting finding of this study is that, when adjusting for confounders, three categories of children showed statistically significant increased odds of positive anti-SARS-CoV-2 IgG antibodies: children previously positive to a nasopharyngeal swab (AdjOR 15.41, 95%CI 3.44–69.04, *p* < 0.001), cohabitant with a person with an history of a previous positive nasopharyngeal swab (AdjOR 9.95, 95%CI 5.35–18.52, *p* < 0.001), and with a foreign citizenship (AdjOR 2.4, 95%CI 1.05–5.70, *p* = 0.002). On the one hand, these results confirm an intrafamilial model of transmission for SARS-CoV-2, as reported by previous studies, which found anti-SARS-CoV-2 IgG antibodies in approximately half of the children exposed to a household contact [30]. On the other hand, the last association is in contrast with previous observations in adult cohorts showing similar prevalence of the infection between Italian citizens and immigrants [31].

As a limitation of this study, the pragmatic approach of enrolling children accessing the hospital for a blood sample was based on ethical considerations, but may have selected our sample toward children with more comorbidities, which, in some cases, may have been more protected by their family, thus having a lower risk of contracting the infection. This, together with our exclusion criteria, may have led us to slightly underestimate the real seroprevalence in the general population of children. However, since the same selections methods were used in the three time points, this should not have affected the comparison among the three time periods. Data collection may also have suffered from a possible recall bias in the information provided by parents. Finally, the sample size was not entirely adequate in detecting a small change in a low prevalence rate, as observed in the study; however, it is sufficient to rule out a clinically significant difference in seroprevalence rate over the three time periods. We aim at conducting other rounds of this study to further document the trend in the seroprevalence rate of children over time. In parallel, further studies should investigate the kinetics of humoral immune response to SARS-CoV-2 infection in children, as well as the cellular response, in order to identify new potential markers of previous SARS-CoV-2 infection such as SARS-CoV-2-specific CD4+ T cells or SARS-CoV-2 receptor-binding domain (RBD)-specific memory B-cells, which have been shown to remain stable even 12 months after the infection in adult patients [32].

In conclusion, no significant differences in the anti-SARS-CoV-2 trimeric Spike protein IgG antibodies prevalence were found in the pediatric population of our region across the second and the third wave of the pandemic, supporting previous observations of a weak and/or quickly volatile antibody response to SARS-CoV-2 in children. In a multivariate analysis, a previous infection from SARS-CoV-2, a previously infected cohabitant, and foreign citizenship were the three factors showing increased odds of positive anti-SARS-CoV-2 IgG antibodies. Data from our study could be relevant for policy makers to plan infection control strategies and may affect the discussion on the which priority to give to the opportunity of vaccinating children younger than 12 [33,34]. On one hand, COVID-19 disease is rarely severe in children, and it is uncertain to what extent IgG antibodies reflect an immunological response to SARS-CoV-2; on the other hand, this study, which detected a seroprevalence rate of only 10–12%, suggests that a large proportion of them may still be susceptible to the infection and may act as silent transmitters, which would likely make difficult a full control of future outbreaks based on adult and adolescent vaccination alone [35].

## Figures and Tables

**Table 1:** Children characteristics in the three time periods.

	January–February N = 217 n (%)	April–May N = 208 n (%)	June–July N = 169 n (%)	*p*-Value
Sociodemographic characteristics				
Sex				
Female	101 (46.5)	99 (47.6)	77 (45.6)	0.92
Male	116 (53.5)	109 (52.4)	92 (54.4)	0.92
Age, median [IQR; min–max]	11 [7–13; 0–17]	11 [7–14; 0–18]	11 [6–13; 0–18]	0.26
Age as category				
<5 years	44 (20.3)	40 (19.2)	39 (23.1)	0.64
5–11 years	69 (31.8)	70 (33.7)	64 (37.9)	0.45
12–18 years	104 (47.9)	98 (47.1)	66 (39.1)	0.17
Citizenship				
Italian	197 (90.8)	196 (94.2)	152 (89.9)	0.26
Foreign	20 (9.2)	12 (5.8)	17 (10.1)	0.26
Children cohabitant with a healthcare worker	27 (12.4)	28 (13.5)	19 (11.2)	0.81
Comorbidity, any	108 (49.8) ^1^	79 (38.0)	66 (39.1)	0.03
Syndromes or genetic disorders	23 (10.6)	28 (13.5)	25 (14.8)	0.44
Autoimmune diseases	21 (9.7) ^1^	8 (3.8)	6 (3.6)	0.01
Obesity/overweight	13 (6.0)	13 (6.2)	6 (3.6)	0.45
Preterm	8 (3.7)	8 (3.8)	4 (2.4)	0.71
Asthma or allergic disease	8 (3.7)	3 (1.4)	4 (2.4)	0.33
Type 1 Diabetes Mellitus	4 (1.8)	3 (1.4)	6 (3.6)	0.38
Cerebral palsy/disability/other neurological disease	4 (1.8)	4 (1.9)	5 (3.0)	0.73
Other	13 (6.0)	7 (3.4)	6 (3.6)	0.34
More than one comorbidity	14 (6.5)	5 (2.4)	4 (2.4)	0.06
No comorbidities	109 (50.2) ^1^	129 (62.0)	103 (60.9)	0.03
Nasopharyngeal swab				
Children ever tested with a nasopharyngeal swab *	148 (68.2)	148 (71.2)	125 (74.0)	0.46
Children ever tested and positive	6/142 (4.1)	7/148 (4.7)	3/122 (2.4)	0.59
Timing (for children ever tested and positive)				
<3 months before	5/6 (83.3)	2/7 (28.6)	1/3 (33.3)	0.13
3–6 months before	1/6 (16.7)	4/7 (57.1)	2/3 (66.7)	0.29
>6 months before	0/6 (0.0)	1/7 (14.3)	0/3 (0.0)	>0.99
Children cohabitant with a person with a history of a previous positive nasopharyngeal swab **	28 (12.9)	36 (17.3)	24 (14.2)	0.43
Children tested by nasopharyngeal swab	28/28 (100) ^1^	28/36 (77.8)	22/24 (91.7)	0.02
Resulting positive	4/28 (14.3)	6/28 (21.4)	2/22 (9.1)	0.54
Results of anti-SARS-CoV-2 Spike protein IgG antibodies				
Positive	24 (11.1)	32 (15.4)	20 (11.8)	0.37

Notes: * Children with a nasopharyngeal swab in the previous 14 days were excluded. ** Children with a history of contact with a positive case in the previous 14 days were excluded. ^1^ Statistically significant *p* value (Bonferroni adjusted *p* < 0.05) in the comparison January–February vs. April–May. No statistically significant differences were found in the comparison April–May vs. June–July, nor January–February vs. June–July.

**Table 2:** Subgroup analysis by results of a previous nasopharyngeal swab.

	January–February n/N (%)	April–May n/N (%)	June–July n/N (%)	*p*-Value
Children with a previous positive nasopharyngeal swab *				
Positive anti-SARS-CoV-2 Spike protein IgG antibodies	6/6 (100)	4/7 (57.1)	3/3 (100)	0.17
Children with a previous negative nasopharyngeal swab *				
Positive anti-SARS-CoV-2 Spike protein IgG antibodies	16/142 (11.3)	17/141 (12.1)	15/122 (12.3)	0.96

Notes: * Children with a nasopharyngeal swab in the previous 14 days were excluded.

**Table 3:** Characteristics of children presenting anti-SARS-CoV-2 Spike protein IgG antibodies vs. children not presenting anti-SARS-CoV-2 Spike protein IgG antibodies.

	Anti-SARS-CoV-2 Spike Protein IgG Antibodies		
	Positive N = 76 n (%)	Negative N = 518 n (%)	*p*-Value
Sociodemographic characteristics			
Sex			
Female	38 (50.0)	239 (46.1)	0.61
Male	38 (50.0)	279 (53.9)	0.61
Age, median (IQR; min–max)	11.0 (6.0, 15.0; 0–18)	11.0 (7.0, 14.0; 0–17)	0.49
Age as category			
<5 years	18 (23.7)	105 (20.3)	0.49
5–11 years	22 (28.9)	181 (34.9)	0.30
12–18 years	36 (47.4)	232 (44.8)	0.67
Citizenship			
Italian	63 (82.9)	482 (93.1)	0.005
Foreign	13 (17.1)	36 (6.9)	0.005
Children cohabitant with a healthcare worker	10 (13.2)	64 (12.4)	0.99
Comorbidity, any	38 (50.0)	215 (41.5)	0.16
Syndromes or genetic disorders	12 (15.8)	64 (12.4)	
Autoimmune diseases	4 (5.3)	31 (6.0)	>0.99
Obesity/overweight	5 (6.6)	27 (5.2)	0.62
Preterm	5 (6.6)	15 (2.9)	0.096
Asthma or allergic disease	1 (1.3)	14 (2.7)	0.71
Type 1 Diabetes Mellitus	2 (2.6)	11 (2.1)	0.68
Cerebral palsy/disability/other neurological disease	2 (2.6)	11 (2.1)	0.68
Other	2 (2.6)	24 (4.6)	0.56
More than one comorbidity	5 (6.6)	18 (3.5)	0.19
No comorbidities	38 (50.0)	303 (58.5)	0.46
Nasopharyngeal swab			
Children ever tested with a nasopharyngeal swab *	61 (80.3)	360 (69.5)	0.05
Children ever tested * and positive	13/61 (21.3)	3/360 (0.8)	<0.001
Timing (for children ever tested and positive)			
<3 months before	6/13 (46.2)	2/3 (66.7)	>0.99
3–6 months before	7/13 (53.8)	0/3 (0.0)	0.21
>6 months before	0/13 (0.0)	1/3 (33.3)	0.19
Children cohabitant with a person with a history of a previous positive nasopharyngeal swab **	41 (54.0)	47 (9.1)	<0.001
Children tested by nasopharyngeal swab	35/41 (85.4)	43/47 (91.5)	0.50
Resulting positive	9/35 (25.7)	3/43 (7.0)	0.03
Anti-SARS-CoV-2 Spike protein IgG antibodies titre (BAU/mL) (IQR; min–max)	201 (74.1–382.2; 33.9–3900)	-	-

Notes: * Children with a nasopharyngeal swab in the previous 14 days were excluded. ** Children with a history of contact with a positive case in the previous 14 days were excluded.

**Table 4:** Multivariate logistic regression of factors associated with positive anti-SARS-CoV-2 IgG antibodies (n = 594).

	AdjOR (95%CI)	*p*-Value
Sex		
Female	1.12 (0.63–2.00)	0.69
Male	Ref	Ref
Age		
<5 years	1.44 (0.69–2.98)	0.33
5–11 years	1.02 (0.52–1.98)	0.96
12–18 years	Ref	Ref
Citizenship		
Italian	Ref	Ref
Foreign	2.45 (1.05–5.70)	0.002
Children cohabitant with a healthcare worker		
Yes	0.69 (0.28–1.75)	0.44
No	Ref	Ref
Comorbidity		
Syndromes or genetic disorders	1.13 (0.49–2.63)	0.78
Autoimmune diseases	0.79 (0.21–2.96)	0.72
Obesity/overweight	1.47 (0.49–4.40)	0.13
Preterm	1.73 (0.43–7.00)	0.44
Asthma or allergic disease	0.45 (0.05–3.94)	0.47
Type 1 Diabetes Mellitus	1.55 (0.27–8.94)	0.62
Cerebral palsy/disability/other neurological disease	1.39 (0.24–8.01)	0.71
Other	0.62 (0.08–4.45)	0.63
More than one comorbidity	3.07 (0.87–10.82)	0.08
No comorbidities	Ref	Ref
Children ever tested with a nasopharyngeal swab *		
Yes	0.99 (0.50–1.97)	0.28
No	Ref	Ref
Children ever resulted positive to a nasopharyngeal swab *		
Yes	15.41 (3.44–69.04)	<0.001
No	Ref	Ref
Children cohabitant with a person with a history of a previous positive nasopharyngeal swab **		
Yes	9.95 (5.35–18.52)	<0.001
No	Ref	Ref
Anti-SARS-CoV-2 Spike protein IgG antibodies test		
January–February	Ref	Ref
April–May	1.45 (0.73–2.86)	0.29
June–July	1.18 (0.57–2.47)	0.66

Notes: * Children with a nasopharyngeal swab in the previous 14 days were excluded. ** Children with a history of contact with a positive case in the previous 14 days were excluded.

## Data Availability

The data presented in this study are available on request from the corresponding author. The data are not publicly available due to privacy issues.
